# Development of a novel continuous measure of adolescent mental health inspired by the dual-factor model

**DOI:** 10.3389/fpsyg.2022.918894

**Published:** 2022-08-12

**Authors:** Nathan King, Colleen M. Davison, William Pickett

**Affiliations:** ^1^Department of Public Health Sciences, Queen’s University, Kingston, ON, Canada; ^2^Faculty of Applied Health Sciences, Brock University, St. Catharines, ON, Canada

**Keywords:** adolescent, mental health, measurement, Dual-factor Model of mental health, psychopathology, subjective well-being

## Abstract

**Background:**

According to the Dual-factor Model, mental health is comprised of two related constructs: subjective well-being and psychopathology. Combining these constructs can provide a more accurate and comprehensive assessment of adolescent mental health than considering either on its own. The model suggests the need to group mental health into four distinct categories, which does not recognize its potential continuum and adds statistical complexity. In this study, we developed a continuous measure inspired by, and as a complement to, the Dual-factor Model. Our goal was to demonstrate a novel approach to developing a valid measure for use in public health research that captures varying mental health states more accurately than traditional approaches and has advantages over the categorical version.

**Methods:**

Self-report data are from the 2014 Canadian Health Behavior in School-aged Children study (*n* = 21,993). Subjective well-being was measured by combining indicators of life satisfaction, positive affect, and negative affect. Internalized and externalized symptoms scales were combined to measure psychopathology. The continuous dual-factor measure was created by subtracting standardized psychopathology scores from standardized subjective well-being scores. Construct validity was assessed using multivariable linear regression by examining associations between factors known to be associated with adolescent mental health status (demographic characteristics, social and academic functioning, and specific indicators of mental health) and average mental health scores.

**Results:**

The average age was 14.0 (SD = 1.41) years. The continuous mental health score ranged from 5 to 67 [Mean (SD): 50.1 (9.8)], with higher scores indicating better overall mental health. The nature and direction of the associations examined supported construct validity. Being from a more affluent family, and having more supportive relationships with family, peers, teachers, and classmates was associated with greater mental health (Cohen’s d: 0.65 to 1.63). Higher average marks were also associated with better mental health. Average mental health scores were much lower if students reported feeling hopeless or rated their health as fair or poor.

**Conclusion:**

A continuous measure of mental health based on the Dual-factor Model appears to be a comprehensive and valid measure with applications for research aimed at increasing our understanding of adolescent mental health.

## Introduction

According to the World Health Organization, optimal mental health involves the successful performance of mental functioning, resulting in productive activities, fulfilling relationships, and the ability to cope with adversity (World Health Organization, 2004). Traditionally, symptoms of mental illness (e.g., anxiety and depression) have been used to infer the presence or absence of optimal mental health. Asymptomatic youth are considered mentally healthy by this approach, despite having varying levels of mental functioning (Greenspoon and Saklofske, 2001). According to the field of positive psychology the presence of subjective well-being (SWB) (e.g., life satisfaction and positive emotions) is also important to optimal mental health and functioning (Seligman and Csikszentmihalyi, 2000; Keyes, 2017).

**FIGURE 1:**
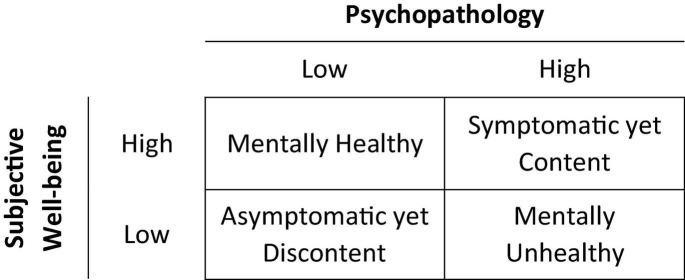
Mental health status groups based on the Dual-factor Model of mental health (Greenspoon and Saklofske, 2001; Wang et al., 2011). The labels used in this study for the mental health groups come from Renshaw and Cohen (2014).

A Dual-factor Model of mental health [also referred to as the Two Continua Model of Mental Health and Illness, the Complete State Model, and the Dual-continua Model (Wang et al., 2011)] was proposed by Greenspoon and Saklofske (2001), as one approach to the assessment of mental health but in composite. The Dual-factor Model states that mental health can be assessed by combining ratings on two dimensions: SWB and psychopathology (Greenspoon and Saklofske, 2001; Wang et al., 2011). SWB is defined according to the hedonic tradition as feeling good about one’s life, and psychopathology refers to the presence of internalized or externalized symptoms and behaviors associated with mental illness. The resultant two-factor measure depicts four mental health groups with unique mental functioning and treatment needs: (1) “Mentally Healthy” (high well-being and low psychopathology), (2) “Symptomatic yet Content” (high well-being, but high psychopathology), (3) “Asymptomatic yet Discontent” (low well-being despite low psychopathology), and (4) “Mentally Unhealthy” (low well-being and high psychopathology) (Figure 1; Wang et al., 2011). Measures based on the Dual-factor Model are felt to provide more accurate and comprehensive assessments of mental health status than traditional unidimensional measures that focus on specific indicators of well-being or psychopathology. Each group reports unique levels of functioning in domains related to mental health (social, physical, behavioral, and academic), with the highest functioning group (mentally healthy) reporting both low psychopathology and high well-being (Lyons et al., 2012; Suldo et al., 2016). “Asymptomatic yet Discontent” youth who would be identified as mentally healthy using traditional disease-based approaches display reduced functioning compared to the “Mentally Healthy” group (Lyons et al., 2012; Suldo et al., 2016) and similar academic struggles as those with high psychopathology (Smith, 2018). “Symptomatic yet Content” youth who would be categorized as mentally unhealthy based on their psychopathology score display better social functioning and academic engagement (Antaramian et al., 2010), and higher self-worth than the “Mentally Unhealthy” group (Suldo and Shaffer, 2008; Suldo et al., 2011). In other words, being symptom free and feeling good (happy and satisfied with life) equates to optimal mental health and functioning.

While evidence supports the application of a dual-factor measure over traditional approaches, there are disadvantages to grouping mental health into four broad categories. In studies that have operationalized a dual-factor measure, over half to two-thirds of adolescents were labeled mentally healthy (Suldo et al., 2016), ignoring within-group variation, and potentially masking important within-group differences. Compared to a continuous variable, a four-category, nominal outcome also adds statistical and interpretive complexity to common regression approaches, is less easily adopted to advanced statistical applications (e.g., structural equation modeling), and requires a greater sample size to detect meaningful effects (Altman and Royston, 2006).

**FIGURE 2:**
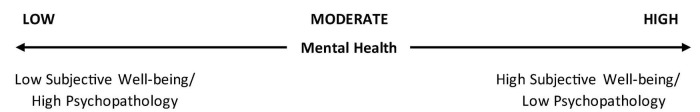
Composite continuous measure of mental health inspired by the Dual-factor Model (Greenspoon and Saklofske, 2001; Wang et al., 2011). Psychopathology = sum of standardized internalized and externalized symptom scores; subjective well-being (SWB) = standardized negative affect score subtracted from sum of standardized life satisfaction and positive affect scores. Each score was then standardized to have a mean of 50 and standard deviation of 10.

The purpose of this study was therefore to develop and test a novel measure of adolescent mental health for use in public health research, based on our adaptation of the Dual-factor Model (King et al., 2021) and in a continuous form (Figure 2). Such a continuous measure could potentially comprehensively and more accurately capture varying mental health states than traditional approaches. It would also possess various statistical advantages over a categorical dual-factor measure. Given the novelty of this measure, we assessed construct validity in its testing.

## Materials and methods

### Overview of research design

**FIGURE 3:**
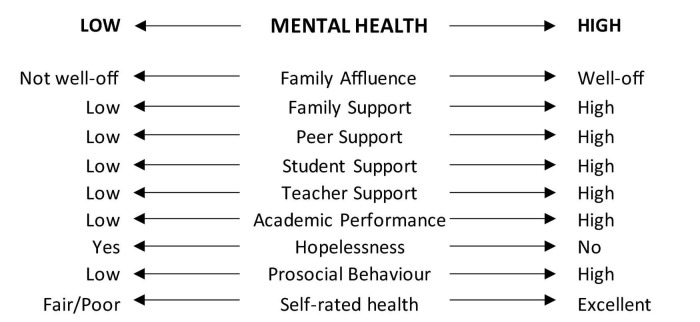
Expected relationships between family affluence, social support, academic functioning, and indicators of mental health, and the composite mental health score.

The steps involved in this research were: (1) development of the continuous measure of mental health based on the Dual-factor Model (Greenspoon and Saklofske, 2001; Wang et al., 2011), combining measures of SWB and psychopathology, and (2) testing of its construct validity (Westen and Rosenthal, 2003), *via* examining measures of association with indicators known to be associated with adolescent mental health (Freeman et al., 2016; McAdam et al., 2018). The latter step included specific indicators of mental health, demographic factors, reports of academic performance, and social support variables. *A priori*, higher mental health scores on our measure were expected to be linearly associated with greater social support, better health, greater academic performance, and greater self-reported family affluence (Figure 3).

### Data source

Data for this study come from the 2014 Canadian Health Behavior in School-aged Children (HBSC) study (Freeman et al., 2016). The sample covers all provinces and territories, with notable exclusions including private and on-reserve schools, home schooled students, and incarcerated students (collectively, < 7% of the eligible student population) (Van Pelt et al., 2015). A self-report general health questionnaire was administered in the classroom, in hardcopy or online formats, that compiled data on health and health-related behaviors from grade 6 to 10 students (typically ages 11 to 15 years old) (Freeman et al., 2016). Participation in the survey required student assent and parental consent (either active and/or passive). Sample weights were developed to ensure national representativeness by grade within province/territory. Ethics approval for the HBSC study was obtained from the Public Health Agency of Canada (REB 2013-0022), and the Queen’s University Ethics Board (GREB TRAQ #: 6010236).

### Variables contributing to the dual-factor inspired measure

The mental health measure was created by combining measures of SWB and psychopathology into a composite score (Figure 2).

#### Subjective well-being

Following precedent (Greenspoon and Saklofske, 2001; Suldo and Shaffer, 2008; Lyons et al., 2012), SWB was measured by combining with equal weighting three separate but related constructs: life satisfaction, positive affect, and negative affect (Diener et al., 1999). Life satisfaction was rated from 0 “*Worst possible life*” to 10 “*Best possible life*” using the Cantril Ladder (Cantril, 1965; Mazur et al., 2018). Positive and negative affect were measured using available HBSC items. A single item (“*I am full of energy*”) from the Positive and Negative Affect Schedule for Children (PANAS-C) (Laurent et al., 2005) was used to measure positive affect. Negative affect was measured using the items “*I often feel lonely*” and “*I often feel helpless*” (five response options: “*Strongly Agree*” to “*Strongly Disagree*”) from the PANAS-C (Laurent et al., 2005) and the Implicit Positive and Negative Affect Test (IPANAT) (Quirin et al., 2009). To achieve equal weighting, life satisfaction, positive affect, and negative affect were standardized to have a mean of 50 and standard deviation of 10. A *SWB score* was calculated by summing standardized life satisfaction and positive affect scores, and then subtracting the standardized negative affect score [SWB = (life satisfaction + positive affect) – negative affect] (Diener et al., 1999; Suldo and Shaffer, 2008).

#### Psychopathology

Internalized symptoms were assessed *via* the four-item psychological symptoms subscale of the subjective health complaints scale (range = 0 to 16; α = 0.79) (Hetland et al., 2002; Freeman et al., 2016). Students reported how often they had the following in the past 6 months: “*Feeling low (depressed)*,” “*Irritability or bad temper*,” “*Feeling nervous*,” “*Difficulties in getting sleep*” (five response options: “*Rarely or never*” to “*About every day*”). Externalized symptoms were measured using an overt risk-taking scale, that combined the frequency of engagement (“*None,”* “*Infrequent,”* “*Frequent”*) in the following behaviors: alcohol consumption, lifetime drunkenness history, smoking history, use of alternative tobacco products, physical fighting, caffeinated energy drink consumption, and non-helmet use on a bicycle (α = 0.75) (Kwong et al., 2018). Internalized and externalized symptom scores were also standardized to have a mean of 50 and standard deviation of 10. The *psychopathology (PTH) score* used in the composite mental health measure was calculated by summing the adolescent’s internalized and externalized symptoms scores. Both internalized and externalized symptoms were included because some adolescents are more likely to internalize their distress, while others are more likely to act out and externalize their behaviors (Herpertz-Dahlmann et al., 2013).

### Variables used in the validation test

Construct validity was assessed by examining whether mental health scores differed in the expected direction on variables describing academic functioning, social support, demographic characteristics, and specific indicators of mental health, as illustrated in Figure 3 (Desocio and Hootman, 2004; Freeman et al., 2011; McAdam et al., 2018).

The measures described below have been scrutinized for face validity and shown to have strong construct validity and internal consistency reliability where applicable (Currie et al., 2014).

#### Demographic characteristics

Age was categorized as ≤ 11, 12, 13, 14, or ≥ 15 years old, and youth identified as either “*Male*” or “*Female.*” Relative family affluence was measured using the item: “*How well off do you think your family is?*” (“*Well-off,”* “*Average,”* “*Not well-off”*).

#### Social support

Family and peer support were measured using the Multidimensional Scale of Perceived Social Support (MPSS) (Zimet et al., 1988). Family support was assessed using four items describing whether students believe their family is available and willing to help them in times of need (five response options: “*Strongly disagree*” to “*Strongly agree*”) (range = 0 to 16; α = 0.91) (Freeman et al., 2016). Peer support was measured using a four-item scale (range = 0 to 16; α = 0.92), describing beliefs they have friends they can count on and confide in (five response options: “*Strongly disagree*” to “*Strongly agree*”) (Freeman et al., 2016).

#### Academic functioning

Student support was measured using a 3-item HBSC scale (range = 0 to 12; α = 0.80), capturing students’ perceptions of their peers within the school environment (five response options: “*Strongly disagree*” to “*Strongly agree*”) (Ravens-Sieberer et al., 2009; Freeman et al., 2016). Teacher support was assessed using nine items (range = 0 to 36; α = 0.90) related to students perceptions of their teachers feelings toward them, and how they felt about their teachers (five response options: “*Strongly disagree*” to “*Strongly agree*”) (Freeman et al., 2016). Academic performance was measured using five categories describing average marks in the past year (1 = “*Mostly letter grades below C/below 50%/or level 1*,” 2 = “*Mostly Cs/between 50 and 59%/or level 2*,” 3 = “*Mostly Bs and Cs/between 60 and 69%/or level 3*,” 4 = “*Mostly As and Bs/between 70 and 84%/or level 3 and 4*,” 5 = “*Mostly As/above 85%/or level 4*”).

#### Mental health indicators

Hopelessness (*Yes* or *No*) was assessed with the item: “*During the past 12 months, did you ever feel so sad or hopeless almost every day for 2 weeks or more in a row that you stopped doing some usual activities.*” This item is the first of five in the Youth Risk Behavior Survey (YRBS) suicidality scale, and a strong predictor of clinical depression (Harter and Whitesell, 1996). Prosocial behavior was measured using a 5-item scale (range = 0 to 25) capturing how often youth engage in behaviors that put others before themselves (α = 0.87) (Freeman et al., 2016). Finally, self-rated health status was measured with the item: “*Would you say your health is*…*?*” (“*Excellent*,” “*Good*,” “*Fair*,” or “*Poor*”).

### Analysis

All analyses were conducted using SAS Version 9.4 (SAS Institute, Cary, NC, United States, 2016).

#### Developing the measure

The adolescent’s psychopathology and SWB scores were each standardized to have a mean of 50 and standard deviation of 10. The composite mental health score was then calculated by subtracting the psychopathology score (higher score = higher psychopathology) from the SWB score (higher score = higher well-being). The resulting score was similarly standardized, with higher scores corresponding to better mental health (Figure 2).

#### Testing the measure

Sample weights were applied to all analyses. Average mental health scores were described by available covariates (Figure 3). Continuous covariates were modeled using approximate quartiles based on the sample distribution. Mixed-effects linear regression was used to test for group differences adjusting for age and sex, and clustering by school using random effects. Cohen’s d was calculated as a standardized measure of effect size (values ≥ 0.8 are considered large, and ≥ 1.2 very large) (Sawilowsky, 2009). Assuming a standard deviation of 10 the analysis was 80% powered to detect differences between means of 0.5 to 2.5, two-sided alpha = 0.05. The above analysis was repeated in the “Mentally Healthy” group as defined according to the four-category Dual-factor Model (King et al., 2021), to examine within group variation in average mental health scores.

## Results

The sample used to create the measure (*n* = 21,993) was 53% female with an average age of 14.0 (SD = 1.41) years. Approximately two-thirds of the sample identified as White, one-fifth were born outside of Canada, and over 80% lived in a home with two adults. The composite mental health score ranged from 5 to 67 [weighted Mean (SD) = 50.1 (9.8)], with higher scores indicating better mental health. The distribution was moderately skewed toward poor mental health (skewness = −0.72). Based on the intraclass correlation coefficient of 0.038, 3.8% of the variability in mental health scores could be attributed to school-level factors.

For the analyses examining the mental health score by selected covariates, the sample was restricted to students with complete data [*n* = 18,720 (weighted *n* = 18,867)]. Adolescents that were excluded because of missing data were comparable to those included by age, gender, relative family affluence, and self-rated health status (*p* > 0.05).

Average mental health scores were significantly different by the covariates examined (*p* < 0.001), in the expected direction (*p*-trend < 0.001) (Table 1). Mental health scores were lower in girls and decreased with increasing age. Being in a more supportive and affluent family, and having more supportive relationships with peers, teachers, and other students were associated with greater mental health. Higher marks in school were also associated with better mental health. Average mental health scores were significantly lower if students reported having felt hopeless or rated their health as fair or poor. Differences were most pronounced (Cohen’s d > 1.2) for family support, academic achievement, feelings of hopelessness, and self-rated health.

**TABLE 1:** Description of the 2014 HBSC sample by average mental health score.

Sex	*n*	(%)	Mean	(SD)	*p* *	*p*-trend*	Cohen’s d
Male	8,774	(46.5)	51.6	(8.8)	ref	/	0.30
Female	10,093	(53.5)	48.7	(10.5)	<0.001		
**Age**							
≤11	1,645	(8.7)	54.8	(7.6)	ref	<0.001	ref
12	3,102	(16.4)	53.4	(8.5)	<0.001		0.17
13	3,550	(18.8)	51.6	(9.2)	<0.001		0.38
14	4,303	(22.8)	49.3	(9.7)	<0.001		0.63
≥15	6,266	(33.2)	46.8	(10.2)	<0.001		0.89
**Relative family affluence**							
Well-off	10,701	(56.7)	52.4	(9.0)	ref	<0.001	ref
Average	6,484	(34.4)	48.0	(9.2)	<0.001		0.48
Not well-off	1,682	(8.9)	43.5	(12.1)	<0.001		0.83
**Family support**							
High	5,242	(27.8)	56.0	(7.6)	ref	<0.001	ref
Q2	5,744	(30.4)	52.2	(7.4)	<0.001		0.51
Q3	3,977	(21.1)	47.6	(8.5)	<0.001		1.04
Low	3,904	(20.7)	41.5	(10.0)	<0.001		1.63
**Peer support**							
High	4,830	(25.6)	53.0	(9.6)	ref	<0.001	ref
Q2	3,190	(16.9)	51.2	(9.5)	<0.001		0.19
Q3	6,112	(32.4)	49.8	(9.2)	<0.001		0.34
Low	4,734	(25.1)	46.6	(10.0)	<0.001		0.65
**Student support**							
High	4,762	(25.2)	54.8	(8.1)	ref	<0.001	ref
Q2	4,708	(25.0)	51.9	(8.2)	<0.001		0.36
Q3	5,007	(26.5)	49.0	(9.2)	<0.001		0.67
Low	4,389	(23.3)	44.1	(10.4)	<0.001		1.15
**Teacher support**							
High	4,761	(25.2)	55.9	(7.3)	ref	<0.001	ref
Q2	4,893	(25.9)	52.0	(7.8)	<0.001		0.52
Q3	4,537	(24.1)	48.7	(8.8)	<0.001		0.89
Low	4,675	(24.8)	43.4	(10.7)	<0.001		1.36
**Academic performance**							
A’s/>84%/level 4	6,036	(32.0)	52.4	(8.1)	ref	<0.001	ref
A’s & B’s/70–84%	9,156	(48.5)	50.2	(10.0)	<0.001		0.24
B’s & C’s/60–69%	2,994	(15.9)	46.7	(11.0)	<0.001		0.59
C’s/50–59%	556	(3.0)	43.4	(10.2)	<0.001		0.98
<C’s/<50%/level 1	124	(0.7)	38.7	(10.4)	<0.001		1.47
**Self-rated health status**							
Excellent	5,626	(29.8)	54.9	(8.0)	ref	<0.001	ref
Good	10,160	(53.9)	49.8	(8.9)	<0.001		0.60
Fair/poor	3,081	(16.3)	42.2	(10.3)	<0.001		1.38
**Feelings of hopelessness**							
No	13,889	(73.6)	53.4	(7.4)	ref	/	1.43
Yes	4,978	(26.4)	40.9	(9.9)	<0.001		
**Prosocial behavior**							
High	4,979	(26.4)	52.1	(10.2)	ref	<0.001	ref
Q2	4,530	(24.0)	50.6	(9.5)	<0.001		0.15
Q3	5,353	(28.4)	49.3	(9.5)	<0.001		0.28
Low	4,005	(21.2)	47.8	(9.6)	<0.001		0.43

(1) Values are weighted, (2)*All p-values obtained from mixed effects multivariable linear regression models that adjusted for age and sex, and clustering by school.

Average mental health scores are described in students categorized as mentally healthy according to the categorical Dual-factor Model in Table 2. Within this group average mental health scores consistently differed by the covariates examined (*p* < 0.001), and in the expected direction (*p* < 0.001) (Table 2). The differences in means for each covariate reached moderate to very large in size according to Cohen’s d (0.63 to 1.38) (Sawilowsky, 2009).

**TABLE 2:** Description of the average mental health score by indicators of social support, academic functioning, and self-rated health in “mentally healthy” adolescents according to the categorical dual-factor measure†[weighted *n* = 14,994 (67.6% of the full sample)].

	*n*	(col%)	Mean	(SD)	*p* *	*p*-trend*	Cohen’s d
**Family support**							
High	4,993	(33.7)	58.0	(5.5)	ref	<0.001	ref
Q2	5,173	(34.9)	54.7	(5.2)	<0.001		0.62
Q3	2,903	(19.6)	52.2	(5.3)	<0.001		1.07
Low	1,744	(11.8)	50.2	(5.8)	<0.001		1.38
**Peer support**							
High	4,196	(28.3)	56.8	(6.3)	ref	<0.001	ref
Q2	2,575	(17.3)	55.6	(5.7)	<0.001		0.20
Q3	4,948	(33.3)	53.9	(5.7)	<0.001		0.48
Low	3,128	(21.1)	53.0	(5.8)	<0.001		0.63
**Student support**							
High	4,363	(29.5)	57.6	(5.5)	ref	<0.001	ref
Q2	4,024	(27.2)	55.0	(5.6)	<0.001		0.47
Q3	3,830	(25.9)	53.5	(5.6)	<0.001		0.75
Low	2,554	(17.3)	51.7	(6.2)	<0.001		1.01
**Teacher support**							
High	4,532	(31.4)	57.9	(5.2)	ref	<0.001	ref
Q2	4,175	(28.9)	54.9	(5.4)	<0.001		0.53
Q3	3,323	(23.0)	52.8	(5.7)	<0.001		0.93
Low	2,404	(16.7)	51.4	(6.3)	<0.001		1.13
**Academic performance**							
A’s/>84%/level 4	5,245	(35.6)	55.4	(5.6)	ref	<0.001	ref
A’s & B’s/70–84%	7,157	(48.6)	54.9	(6.1)	<0.001		0.09
B’s & C’s/60–69%	2,014	(13.7)	53.4	(6.6)	<0.001		0.33
C’s/50–59%	277	(1.9)	52.9	(6.2)	<0.001		0.42
<C’s/<50%/level 1	42	(0.3)	51.0	(5.0)	<0.001		0.83
**Self-rated health status**							
Excellent	5,210	(35.0)	57.4	(5.6)	ref	<0.001	ref
Good	8,155	(54.8)	53.9	(5.8)	<0.001		0.61
Fair/poor	1,507	(10.1)	50.8	(5.5)	<0.001		1.19

(1) Values are weighted, (2)*All p-values obtained from mixed effects multivariable linear regression models that adjusted for age and sex, and clustering by school, and (3) †A description of the categorical dual-factor measure is described elsewhere (King et al., 2021).

## Discussion

Inspired by the Dual-factor Model (Greenspoon and Saklofske, 2001; Suldo and Shaffer, 2008; Wang et al., 2011), a novel continuous measure of mental health was developed in a representative sample of Canadian adolescents by combining measures of SWB and psychopathology. In tests of this measure, large and significant differences in mental health scores were identified in the expected direction for all covariates examined, including indicators of social support, academic functioning, global health status, and feelings of hopelessness. This provides evidence in support of the construct validity of this continuous measure. We believe it can be used as a summary indicator of mental health status.

The development of our measure was guided and supported by a contemporary theory of mental health (Greenspoon and Saklofske, 2001; Suldo and Shaffer, 2008). According to the Dual-factor Model, which argues that mental health is comprised of two separate but related constructs, this measure has greater content validity than measures that consider one dimension (Greenspoon and Saklofske, 2001; Suldo and Shaffer, 2008; Wang et al., 2011). By combining the positive and negative dimensions of mental health a more accurate and comprehensive assessment of overall mental health status can be made than if they are considered separately (Wang et al., 2011). By including more information, it may also be a more stable and reliable measure of mental health than relying on a single dimension, potentially reducing random measurement error.

In studies that have operationalized a categorical dual-factor measure, adolescents who scored positively on measures of psychopathology and well-being reported better functioning (e.g., higher grade point average and more social support) than those who scored positively on one dimension only (Suldo and Shaffer, 2008; Antaramian et al., 2010; Kelly et al., 2012). The presence of well-being in addition to the absence of psychopathology is essential to optimal or complete mental health (Wang et al., 2011). On the opposite end of the spectrum, low well-being in the presence of active mental illness is associated with the worst mental health and functioning (Wang et al., 2011). This is consistent with the two ends of the mental health continuum defined by our measure (Figure 2), and our finding of a linear increase in average mental health scores with increasing support and academic functioning.

A potential limitation of the continuous measure is that it cannot distinguish whether suboptimal mental health is related to deficits in well-being or the presence of psychopathology. These two groups might have different etiological factors at work, interventional needs, and risk trajectories. The categorical dual-factor measure may be better suited for identifying the source of adolescents current or future mental health-related difficulties, and the appropriate strategy for intervention (i.e., treating symptoms, or promoting well-being). The continuous measure, however, is well suited for population health research aimed at examining associations between risk and protective factors and levels of adolescent mental health. In this study we consistently identified significant differences in the expected direction between the continuous measure of mental health and various indicators of mental functioning, suggesting that different levels of mental health are accurately captured across the full continuum of possible scores. Further, the two groups that cannot be differentiated with the continuous measure (symptomatic yet content, and asymptomatic yet discontent) report similar academic functioning (Suldo and Shaffer, 2008; Antaramian et al., 2010) and physical health (Suldo and Shaffer, 2008). However, symptomatic yet content youth often report more supportive relationships with family and peers (Antaramian et al., 2010; Kelly et al., 2012), and greater emotional and cognitive engagement at school (Antaramian et al., 2010).

Unlike the categorical dual-factor measure (Greenspoon and Saklofske, 2001; Wang et al., 2011), the continuous version takes advantage of the full range of data. Categorizing mental health ignores within group heterogeneity, potentially leading to an underestimate of effects, and the concealing of non-linearity in the relationships of interest (Altman and Royston, 2006). In studies operationalizing a categorical measure the majority of adolescents are grouped as mentally healthy (as high as 67%) (Antaramian et al., 2010). This means that the majority of the distribution of the continuous measure, and a potentially wide range of mental health is collapsed into this one group. In a secondary analysis we examined whether average mental health scores differed by indicators of academic and social functioning, and self-rated health in the mentally health group defined by the categorical dual-factor model (King et al., 2021). Results showed significant variability in functioning within this group. These findings suggest that significant variability in mental health status is masked by the categorical measure, and the continuous measure is able to differentiate levels of mental health within “mentally healthy” adolescents, and across the full range of the distribution. This appears to be a distinct advantage of the continuous dual-factor measure over its categorical counterpart.

The continuous measure also provides a more viable option for smaller studies with limited power. In studies categorizing mental health, one or more of the mental health groups is represented by a small percentage of the sample (<12% is common) (Suldo and Shaffer, 2008; Antaramian et al., 2010; Kelly et al., 2012). In smaller samples it becomes difficult to impossible to run a statistical analysis and/or generate meaningful results on a category with such small numbers. Finally, irrespective of sample size a continuous measure is more easily modeled in advanced statistical applications than a four-category nominal variable. In standard path analysis for example there is an assumption that variables are measured on an interval scale (O’Rourke and Hatcher, 2013). Fitting a model with a nominal categorical variable adds complexity in all stages of the analysis from conceptualization to interpretation of the output.

Future studies combining the two dimensions of mental health into a continuous measure might consider different weighting, based on findings that symptomatic yet content youth on average appear to report slightly greater mental functioning than asymptomatic yet discontent youth (i.e., giving more weight to SWB) (Suldo and Shaffer, 2008; Antaramian et al., 2010; Kelly et al., 2012). Future studies might also consider whether internalized and externalized symptoms should be treated equally, as they were in this study. Internalized symptoms (e.g., anxiety and depression) may have a stronger correlation with overall mental health status than externalized symptoms for example, which could be accounted for in the development of the measure.

Strengths of this study included the use of a large, representative sample of Canadian adolescents to develop and test a novel measure of adolescent mental health, making the results generalizable to a wide population. Well-validated scales were used to measure life satisfaction, psychopathology and the covariates included in the tests of construct validity (Currie et al., 2014). This study provides a practical approach to developing a valid, contemporary measure of mental health status that can be applied in future research studies. Several limitations also warrant comment. Because this was a secondary analysis, limited data were available for measuring well-being and psychopathology. The use of validated scales rather than individual items to measure the indicators of well-being, particularly positive and negative affect, could further strengthen the measure. Similarly, the inclusion of other measures of externalized symptoms (e.g., aggression, impulsivity, and hyperactivity) could also strengthen the measure. The use of self-report data likely resulted in some social desirability bias, especially for externalized symptoms.

## Conclusion

In this study we demonstrated a novel approach to developing a valid measure of adolescent mental health status that is more accurate and comprehensive than traditional unidimensional measures and has advantages over the categorical version. A continuous measure of adolescent mental health based on the Dual-factor Model appears to be construct valid and has applications for public health research aimed at increasing our understanding of the factors and circumstances that influence adolescent mental health.

## Data availability statement

The data analyzed in this study is subject to the following licenses/restrictions: Data cannot be shared publicly due to Health Behavior in School-aged Children (HBSC) restrictions. However, data are available for researchers who meet the criteria for access to confidential data. Requests to access these datasets should be directed to dmc@hbsc.org.

## Ethics statement

Ethics approval for the HBSC study was obtained from the Public Health Agency of Canada (REB 2013-0022), and the Queen’s University Ethics Board (GREB TRAQ #: 6010236). Participation was voluntary, and written informed consent (explicit or implicit depending on local protocol) was obtained from school administrators, parents, and participating students.

## Author contributions

NK, CD, and WP contributed to the design and conception of the study. NK performed the statistical analysis and wrote the first draft of the manuscript. All authors revised the manuscript and approved the submitted version.
